# Clinical dental students’ knowledge regarding proper dental settings for treating patient during COVID-19: A cross-sectional study

**DOI:** 10.12669/pjms.37.2.3768

**Published:** 2021

**Authors:** Fahd A Aljarbou, Sundus M Bukhary, Abdullah U Althemery, Abdullah S Alqedairi

**Affiliations:** 1 Fahd A Aljarbou, BDS, MS. Associate Professor, Department of Restorative Dental Sciences, Division of Endodontics, College of Dentistry, King Saud University, Riyadh 11545, Saudi Arabia; 2 Sundus M Bukhary, BDS, MSc. Lecturer, Department of Restorative Dental Sciences, Division of Endodontics, College of Dentistry, King Saud University, Riyadh 11545, Saudi Arabia; 3 Abdullah U Althemery, PhD. Assistant Professor, Department of Clinical Pharmacy, College of Pharmacy, Prince Sattam Bin Abdulaziz University, Al-Kharj, Saudi Arabia; 4 Abdullah S Alqedairi, BDS, MS. Associate Professor, Department of Restorative Dental Sciences, Division of Endodontics, College of Dentistry, King Saud University, Riyadh 11545, Saudi Arabia

**Keywords:** COVID-19, Dental students, Knowledge, Multicenter, Pandemic, Saudi Center for Disease Control (Saudi CDC)

## Abstract

**Objectives:**

To investigate the clinical dental students’ knowledge on the proper dental setting during COVID-19.

**Methods:**

Using multicenter cross-sectional study, a 10-items questionnaire was distributed in April 2020 among senior dental students from two dental schools. It comprised three main domains: demographic questions, questions investigating student’s access to the proper recommendations of dental settings during COVID-19 and the specific recommendations questions.

**Results:**

The questionnaire was sent to 654 clinical dental students and the total number of respondents were 267 representing a response rate of 40.83%. The overall knowledge mean was low (1±0.92). Gender was not a statistically significant factor affecting the knowledge score (P > 0.05). Most of the participants never visited the Saudi Center for Disease Prevention and Control (Saudi CDC) website and received no guidance or advice regarding proper dental practices during COVID-19. A statistically significant relationship was observed between the mean knowledge score and access to knowledge variables (P < 0.05).

**Conclusion:**

It was noted that clinical dental students have low knowledge on the proper dental settings during COVID-19 pandemic that was recommended by the Saudi CDC guidelines and they must be equipped with adequate knowledge from reliable sources to overcome their insufficiencies such as a well-structured and dynamic curriculum.

## INTRODUCTION

The COVID-19 pandemic has affected many aspects of daily life. It is caused by the SARS-CoV-2 virus and originated in Wuhan, China, in December 2019, as multiple cases presenting with viral pneumonia emerged.^1^ The symptoms can be serious and lead to death.^2^ Soon after, widespread infection occurred in different parts of the world in the form of pandemic disease. As of 25 June 2020, there have been 9,296,202 confirmed cases of COVID-19 globally, including 479,133 deaths, reported to World Health Organization (WHO).^3^ To reduce the chances of being infected or spreading COVID-19, WHO posted an advice for public on how to protect yourself and others and highlighted the importance of social distancing, washing hands, avoiding going to crowded places and self-isolation even with minor symptoms.^4^ The health profession was acutely involved with this virus including dentistry. The effect on dental practices generally started as complete closure then moved towards strict compliance with a proper protocol and hopefully, open scheduling in the near future especially when a proved vaccine works.^5^ Dental schools went through what dental practices experienced and they count on staff application of the suggested protocols including students. International and local authorities made the recommendations readily available on their official websites and social accounts to allow medical professionals to be continuously updated and this would improve the preparedness to treat during such a pandemic.^6^,^7^

The Saudi Center for Disease Prevention and Control (Saudi CDC) represents the local authority to control and contain the virus. The center was established in April 2013 by the Council of Minister Resolution to help in disease prevention and control aspects fulfilling the 2030 Vision in Health Promotion. Since then, the center has taken the lead in controlling communicable and non-communicable diseases to promote the health of individuals, making it an important health-monitoring facility in the kingdom. The center’s website content is frequently updated based on new findings and in collaboration with the Center for Disease Prevention and Control (CDC), which is a rich and informative source of health-related information.^8^

One of the main concerns in the COVID-19 pandemic is the speed of transmission, and person-to-person transmission was confirmed.^9^ Daily exposure of dentists to saliva places them at a high risk because viable virus was detected in saliva.^10^ Moreover, aerosols and droplets generated during dental procedures and time required for each procedure can play a significant role in spread of infection.^11^,^12^ Many knowledge gaps persist, particularly regarding the exposure and possible infection of health workers.^13^ The Occupational Safety and Health Act (OSHA) published guidance on preparing workplaces for COVID-19, and they placed dentists at a very high risk category to known or suspected cases.^14^ They even recommended specific engineering and administrative controls while ensuring a safe work practice and proper adherence to the use of personal protective equipment. Dental schools got prepared to run the clinics after following these protocols and efforts were made to spread the knowledge among professional workers. Students are part of the efforts against COVID-19 spread but they used to get their knowledge from curricular activities. This might be a gap in spreading any knowledge from unprecedented situation.

To date, few studies have been published to assess the dental students’ knowledge toward the recommended dental settings during COVID-19.^15^,^16^ However, no similar studies have been done in Saudi Arabia. Therefore, this study aimed to investigate the clinical dental students’ knowledge on the proper dental settings during COVID-19 according to the Saudi CDC guidelines using multicenter cross-sectional study in Riyadh, Saudi Arabia.

## METHODS

The multicenter cross-sectional study was conducted among male and female fourth- and fifth-year undergraduate dental students. The dental schools included in the study were from King Saud University (KSU), a governmental school, and Riyadh Elm University (REU), a private dental school. The study was reviewed ethically by the Institutional Review Board (IRB) committee at the College of Medicine at King Saud University, Saudi Arabia (E-20-4847) and by the College of Dentistry Research Centre (CDRC), King Saud University, Saudi Arabia (FR 0539). The study was conducted during the lockdown on the cities of Riyadh due to the coronavirus pandemic.

An English-language electronic-based self-administered questionnaire was structured and tested among a random sample of dental students at King Saud University (N=20) and then modified according to the feedback obtained. The questionnaire comprised three main domains: demographic questions, access to knowledge questions and knowledge questions. Knowledge domains questions consisted of six questions about the dental settings after COVID-19 pandemic and it was based on The Saudi Center for Disease Prevention and Control (Saudi CDC) without modifications.^8^ Electronic mail addresses of all fourth and fifth-year dental students were collected from the student directories of both dental schools and formal invitations were sent to participate in the study by answering the attached electronic-based questionnaire. All participants voluntarily provided their informed consent upon completion of the questionnaire.

The questionnaire consisted of six knowledge questions carrying one mark each. Each correct answer counted for one point. The three domains were presented in Table-I and II. A chi-square goodness of fit test was utilized to test the frequencies of all categorical data for significant differences. Comparisons of average means were calculated using Student’s t-test. The data were analyzed using Statistical Package for Social Studies (SPSS 22; IBM Corp., New York, NY, USA). A *p*-value < 0.05 was considered statistically significant.

**Table-I:** The frequencies and percentages of the participants in the demographic and access to knowledge questions (N=267).

*Variable*	*No. (%)*
***Participants***	
Females	153 (57.30)
Males	114 (42.70)
***Students Attending Dental School:***	
Public Sector	143 (53.56)
Private Sector	124 (46.44)
***Visited The Saudi Center for Disease Prevention and Control website:***	
Yes	66 (24.72)
No	201 (75.28)
***Received any guidance or advice regarding proper dental practices during COVID-19:***	
Yes	52 (19.48)
No	215 (80.52)
***Type of resource utilized if there was guidance:***	
Official (primary or tertiary)	30 (11.24)
Social media	22 (8.24)
No resources utilized	215 (80.52)

**Table-II:** Frequencies and percentages of knowledge variables of the participants. Correct answers are bolded and underlined.

*Variable*	*No. (%)*
***When grouping patients with cough or other respiratory symptoms in a room, what is the recommended minimum spatial distance in meters?***	
0.5	3 (1.2)
1.0	76 (28.46)
1.5	161 (60.30)
Don’t know	27 (10.11)
***When performing an aerosol-generating procedure in patients with COVID-19 in a negative pressure room, what is the minimum required number of air changes per hour?***	
4	23 (8.61)
8	14 (5.24)
12	11 (4.12)
Don’t know	219(82.02)
***When performing an aerosol-generating procedure in patients with COVID-19 in a facility with a natural ventilation room, what is the minimum required quantity of air in liters/second/patient?***	
120	16 (5.99)
140	21 (7.87)
160	9 (3.37)
Don’t know	221 (82.77)
***According to the Isolation Precautions for COVID-19 Infection control measures in symptomatic confirmed patients , how many negative respiratory sample/s is/are needed to discontinue designated facility isolation precautions?***	
1	13 (4.87)
2 at 24 hours apart	34 (12.73)
2 at 48 hours apart	37 (13.86)
I don’t know	183(68.54)
***According to Isolation Precautions for COVID-19 Infection control measures in asymptomatic confirmed patients , how many negative respiratory sample/s is/are needed to discontinue designated facility isolation precautions?***	
1	16 (5.99)
2 at 24 hours apart	27 (10.11)
2 at 48 hours apart	39 (14.61)
I don’t know	185(69.29)
***According to Isolation Precautions for COVID-19 Infection control measures in suspected cases , what is the proper initial test?***	
Body temperature	141 (52.81)
COVID respirator	21 (7.87)
COVID PCR	44 (16.48)
I don’t know	61 (22.85)

## RESULTS

In total, 267 out of 654 clinical dental students from both schools who received the questionnaire completed the survey. The numbers of females and males were 153 (57.3%) and 114 (42.7%), respectively. Most of the participants were from a governmental college (n= 143, 53.56%), with a response rate of 57.2%. The remaining participants were from a private college (n=124, 46.44%), with a response rate of 30.7%. Most of the participants had never visited The Saudi Center for Disease Prevention and Control website (n=201, 75.28%) and received no guidance or advice for proper dental practices during the COVID-19 pandemic (n=215, 80.52%). Detailed demographic and access frequencies, as well as the associated percentages of participants, are presented in Table-I.

The overall mean knowledge score was low (1 ± 0.92) out of 6. The frequencies and percentages of knowledge variables are presented in Table-II. Private school students performed significantly better than students in the public sector school (p=0.02). Also, students who had visited the Saudi CDC website performed significantly better than those who did not (p=0.009). Students who had received any guidance or advice regarding COVID-19 performed significantly better than those who did not (*p* = 0.001). The detailed means and standard deviation comparisons of these categorical variables are presented in Table-III.

**Table-III:** Detailed mean knowledge scores with standard deviations based on the access to knowledge variables.

*Variable*	*Average knowledge score (SD)*	*P value*
***Gender:***		
Female	1.13 (0.90)	0.50
Male	1.00 (0.92)	
***Dental School:***		
Public sector	0.81 (0.94)	0.02
Private sector	1.42 (0.95)	
***Visited The Saudi Center for Disease Prevention and Control website***		
Yes	1.08 (0.99)	0.009
No.	0.65 (0.87)	
***Received any guidance/advice regarding proper dental practices during COVID-19***	
Yes	1.12 (0.89)	0.001
No	0.67 (0.89)	
***Type of resource utilized***		
Official (primary or tertiary)	1.13 (0.90)	0.88
Social Media	1.09 (1.11)	

## DISCUSSION

The high-risk occupational exposure of dental care professionals (DCPs) during the COVID-19 global public health crisis necessitated that they strictly follow the national and international published dental precautions to avoid cross-infection between DCP and patients. Furthermore, DCPs must be prepared to properly manage patients and the workplace setting during the COVID-19 pandemic.^14^,^17^ We aimed to investigate the knowledge of clinical dental students on the proper dental setting for treating patient during COVID-19 using a multicenter cross-sectional study in Riyadh, Saudi Arabia. To our best knowledge, this is the first study to assess that aim and investigate the correlated variables in Saudi Arabia.

The research sample represented the accessible population who shares the same characteristics of being senior dental students in Riyadh. There are different methods of distributing an electronic and paper questionnaire including E-mails, social media, websites, mail or in-person. The questionnaires in the present study were distributed among the targeted students using E-mail. Although E-mail response rates exceeding 70% have been recorded, rates of 20% or lower are not uncommon and the most effective survey method is still the in-person survey.^18^ However, the overall response rate in this study was 40.8% and this can be attributed to the good professor/student rapport that motivated many students to participate.

Senior dental students were selected in the present study as questionnaire participants because they will practice at clinics as soon as school resumes its academic activity according to local authority recommendations. Therefore, the students must be fully educated and updated regarding the published dental care and dental workplace guidelines.^8^,^14^ The findings of this study revealed an overall weak knowledge score for senior dental students on proper COVID-19 practices accompanied by their lack of awareness about most of the Saudi CDC website. Moreover, most of the participants received no guidance or advice regarding current proper dental practices from any source. This shortage of knowledge is not limited to the Saudi students but was seen on other parts of the world.^15^ Our findings agree with several previous studies showing limited knowledge regarding Middle East Respiratory Syndrome-coronavirus (MERS-CoV) among healthcare workers in Saudi Arabia.^19^,^20^ Thus, the results highlight the importance of educating dental students about formal dental care guidelines before resuming their dental work. This gap of knowledge can be attributed to deficiencies in effectiveness of dental education curriculums. A call for amendments in dental teaching should take place to seal this gap with more effective blended learning that include dynamic topics such as crisis management during a health or natural disasters.^21^

The findings of the present study showed no significant difference in the average score between students who had received information from professional resources and those who had received the information from social media. This can be explained by the presence of formal professional accounts on social media where the information and recommendations are likely more accessible and spread readily. Several published studies have highlighted the role of social media as a source of information for MERS-CoV.^22^,^23^

According to the Saudi CDC, a minimum spatial distance of a least one meter should be maintained when grouping patients with cough or other respiratory symptoms in a room to avoid cross-infection.^8^ Most of the participants’ answers were incorrect, revealing weak knowledge about proper patient grouping. The results of our study agree with the findings of a previous study reporting low awareness of dental students toward spatial distance when managing MERS-CoV.^24^

Evidence has shown that the bioaerosols generated during dental procedures can spread infection through contaminated aerosols and droplets.^11^,^12^ Dental handpieces, ultrasonic scalers, air polishing, air abrasion units, lasers and air/water syringes were shown to produce the most visible aerosols.^25^ The contaminated droplets remain airborne for 30 minutes to two hours.^26^ Thus, DCP and people within the office are exposed to an infinite number of microorganisms per cubic meter.^27^ The Centers for Disease Control and Prevention (CDC) stated clear guidelines for health workers performing aerosol-generating procedures. Emergency dental care for a patient with suspected or confirmed COVID-19 should be performed in an isolation room with negative pressure with at least 12 air changes per hour; if natural ventilation is used, air changes should be at least 160 liters/seconds/patient.^28^ In the current study, less than 5% of the dental students responded correctly to the questions related to the number of air changes in both a negative pressure room and natural ventilation. The findings of this study agree with those of a previous study reporting the poor knowledge of dental professionals toward additional precautions while aerosol-generating procedures were performed during the MERS-CoV.^29^

According to the Isolation Precautions for COVID-19 Infection control measures, the initial test to be carried out in suspected cases is COVID polymerase chain reaction (PCR) that detects antibodies produced in response to infection.^8^ In the present study, only 16.48% of the participants knew the correct initial test in suspected cases. Furthermore, less than 15% of the participants knew the policy to discontinue the designated facility isolation precautions. According to the Saudi CDC, for both symptomatic and asymptomatic confirmed cases, retesting must be performed if the patient has recovered clinically. They reported that, if a patient’s result is positive, the test should be repeated every 72 hours. To discontinue hospital isolation precautions, two negative samples 24 hours apart are required. After discharge, the patients are recommended to continue 14 days of home isolation. Thus far, no comparable data are available to assess the knowledge of dental students in this situation.

In the current study, gender did not affect the knowledge average score. The result agrees with the findings of a previous study that found that gender does not correlate with the knowledge and attitude of dental facilities toward COVID-19 disease.^30^ The results of this study showed that students from private schools performed significantly better than those in government schools. One explanation could be the significant and strong presence and participation of the administrative staff and health professionals from the private school in the social media. Although social media tools may present potential risks to health care providers and receivers in regard to the violation of patient privacy and personal–professional boundaries, licensing or legal issues and distribution of poor-quality information,^31^ these tools can be used to improve or enhance student and patient education, patient care and professional networking.^31^

### Limitation of the study

It was conducted in two dental schools in the central region of Saudi Arabia at a specific time. Thus, the results cannot be generalized to other dental institutes of Saudi Arabia or even out of the country. Moreover, the questions asked were specific and might not represent the full student’s knowledge on the proper dental setting protocols suggested by the local authorities. However, the findings emphasized the need of educational interventions to improve the infection control measures and dental settings after COVID-19.

## CONCLUSIONS

It was noted that clinical dental students have low knowledge on the proper dental settings during COVID-19 pandemic that was recommended by the Saudi CDC guidelines and they must be equipped with adequate knowledge from reliable sources to overcome their insufficiencies such as a well-structured and dynamic curriculum.

### Authors` Contribution:

**FAA** conceived, designed, reviewed the literature and did data collection & editing of manuscript and takes the responsibility and is accountable for all aspects of the work in ensuring that questions related to the accuracy or integrity of any part of the work are appropriately investigated and resolved.

**SMB and AUA** collected and interpreted data and did statistical analysis and wrote up results and discussion.

**ASA** did data collection, statistical analysis and the methodology write up, revised the final manuscript before submission.
